# Hospitalization for pertussis: profiles and case costs by age

**DOI:** 10.1186/1471-2334-5-57

**Published:** 2005-07-11

**Authors:** Judith A O'Brien, J Jaime Caro

**Affiliations:** 1 Caro Research Institute, 336 Baker Avenue, Concord, MA, USA; 2 Division of General Internal Medicine, McGill University, 687 Pine Avenue, West, Montreal, Quebec, Canada

## Abstract

**Background:**

Pertussis, a highly contagious respiratory illness, affects people of all ages and can have serious clinical consequences. It has been reported that from 1997–2000, 20% of all pertussis cases required hospitalization in the US. This analysis examined demographics, case fatality rate, resource use and costs of hospital care related to pertussis by age.

**Methods:**

ICD-9 codes (033.0, 033.9) were used to identify cases of pertussis in hospital discharge databases from roughly 1,000 US hospitals in 4 states (California, Florida, Maryland, Massachusetts). Data from 1996–1999 were examined by age group. Separate analyses were done for infants (<1 year) and children (1–11 years); however, adolescent and adult cases were combined into one group (12+ years), due to the small number of cases. Databases were used to determine demographics, health service utilization and care costs. Cost estimates include accommodations, ancillary and physician services, reported in 2002 US$.

**Results:**

Of the 2,518 cases identified, 90% were infants. The inpatient case fatality rate was <1%. Of survivors, 99% were discharged home (6% with home health care); 1% required further sub-acute inpatient care. For the 2,266 infants, the mean LOS was 6 days at a cost of $9,586 per stay. Children (n = 191) had a mean LOS of 3.7 and cost of $4,729; adolescents/adults (n = 61, mean age 40 years) stayed on average 3.4 days with a cost of $5,683 per hospitalization.

**Conclusion:**

Infants are responsible for the bulk of hospitalizations and generate higher inpatient costs. Costly hospital care occurs, however, in patients with pertussis at all ages.

## Background

*Bordetella pertussis *is a highly contagious respiratory illness that can have serious clinical consequences. It is endemic in the United States (US) and every 3 – 5 years an outbreak occurs [1]. It is most commonly thought of as a childhood disease, not ranking high on the list of differential diagnoses for adolescents or adults presenting with respiratory symptoms. While this may be understandable as the reported incidence of pertussis declined by 99.6% between the mid-1930s to 1970s [2] due to effective childhood vaccination programs, clinicians need to be aware that pertussis continues to be a problem that is on the rise. Since the late 1980s, the incidence of reported pertussis has been increasing in most age groups in the US, particularly among adolescents and adults [1,3-8]. The exception was among young children where the reported incidence has remained stable and likely reflects protection resulting from pertussis vaccinations administered as recommended [9]. In 2002, the greatest number of pertussis cases (n = 9,771) since 1964 were reported in the US resulting in an incidence of 3.4 per 100,000. In the period from 1990 to 2001, a 400% increase in the adult incidence was noted [10] and this is likely an underestimation [11]. Infants continue to be the most vulnerable group. In 2002, 21% of all reported cases were infants too young (age < 6 months) to have started the recommended vaccination program [9]. Nationwide in that year, the reported incidence for infants less than six months old was 108.8 compared to 1.2 per 100,000 among those aged 20 years and older [9]. According to the Centers for Disease Control (CDC), pertussis is the only disease for which childhood vaccination is recommended that has had an increase in incidence in the past 20 years [10].

There is a common misconception vaccinating children for pertussis protects them forever. That is not the case. Immunity achieved through childhood vaccinations starts to diminish after three years with little protection left after twelve years [12-15]. Boosters are not recommended after seven years of age [2] so older adolescents and adults are vulnerable. Furthermore, those who contract pertussis later in life may go undiagnosed, but their sustained cough spreads the infection among family, friends, associates and health care workers [2,6,7,13,15-17].

Although outpatient management with antibiotics, usually erythromycin, is the expected treatment for pertussis [1], hospitalization does occur for those with complications [18-21]. According to the CDC, from 1997 – 2000, 20% of all patients with reported pertussis required hospitalization. This rate was much higher (63%) in infants younger than 6 months [22]. The objective of this analysis was to examine cases of pertussis admitted to US hospitals over a period of four years. Case fatality rates, complications, resource use and inpatient care costs by age group were studied.

## Methods

The cost of hospital care was determined by analyzing inpatient resource use and costs reported in all payer discharge databases from four states representing different areas of the US. Discharge and cost data from roughly 1,000 hospitals in California, Florida, Massachusetts and Washington State for the years 1996 through 1999 were analyzed to identify cases of pertussis, defined as those admitted with an International Classification of Diseases – 9^th ^Revision – Clinical Modification (ICD-9) code of either 033.0 or 033.9 [23] as the principal diagnosis for all age groups. These databases contain merged discharge-level demographic, clinical and economic data for all hospital discharges reported in each state within a given year for patients of all ages. Data on length of stay (LOS), charges incurred, source of admission and discharge disposition were abstracted for each patient. Secondary diagnosis codes reported in the databases were examined for complications of pneumonia in whooping cough (ICD-9 code: 484.3), other pneumonias (ICD-9 codes: 480.0–484.1, 484.4–486), convulsions (ICD-9 codes: 780.31, 780.38), apnea (ICD-9 codes: 770.8, 786.03), encephalopathy (ICD-9 codes: 348.3, 348.5) and acute respiratory distress, failure or arrest (ICD-9 codes: 518.0, 518.81, 518.82, 786.09, 799.0, 799.1).

The acute care hospital costs reported include all accommodations (e.g., routine, intensive care unit, nursery), ancillary (e.g., pharmacy, laboratory, imaging), and physician services. As costs pertaining to physicians' services are not included in the discharge data, it was necessary to estimate an average cost of inpatient physician services. This estimate was derived by determining the type and frequency of visit or service, applying the appropriate unit cost for each and multiplying it by the proportion of inpatients using that service. Explicit data regarding type of visit and services are not provided; therefore, data elements from the discharge databases providing information pertinent to physician care, such as LOS, procedure codes, special care unit stays, emergency department use prior to admission, admission source and discharge status were used to establish a likely inpatient physician visit/service profile for each age group. The appropriate Physicians' Current Procedural Terminology (CPT-4) code was assigned to each visit, service or procedure [24] in the profile. A unit cost representative of diverse payers was established using fee schedules [25,26] and published payer ratios [27], and applied to each CPT-4 code in the profiles. Where physician fee data from a single state were used (i.e., Florida Medicaid physician fees), state costs were adjusted to national values using published ratios [28].

The costs presented here are meant to reflect the economic value of the resources consumed, regardless of who actually pays for them. They represent only direct medical costs – those associated with the delivery of the health care service itself. Indirect costs, such as those due to a parent's lost wages when caring for a child with pertussis, are not included. All cost estimates are reported in 2002 US dollars. Values from previous years were inflated using rates based on the medical care component of the US Consumer Price Index, supplied by the Federal Bureau of Labor Statistics for the appropriate years [29]. Hospital charges for inpatient accommodations and ancillary services were adjusted to costs using a cost-to-charge ratio. There is no standard or national cost-to-charge ratio for hospital care that is reflective of care provided to the scope of patients included in this analysis – those of all ages and covered by different insurers. In the absence of a standard value, 0.61 was used based on a figure calculated by the Commonwealth of Massachusetts Office of Health Care Finance and Policy for hospitals in Massachusetts. The discharge data from each state was analyzed individually first, then these were combined giving equal weight to each state to avoid states with substantially larger populations from being overrepresented.

Cases were analyzed by age groups defined initially as: infants (aged less than 1 year); children (1 through 11 years); adolescents (12 through 17 years) and adults (age greater than 22 years); however, as the number of adolescent and adult cases was insufficient to analyze as separate groups, they were combined.

Seasonal occurrence was explored in terms of the month of hospitalization, except for Florida that reported only the quarter. Analyses by calendar quarter were also carried out for all cases.

Incidence estimates for the states represented in this analysis, as well as nationwide, for the years 1996 – 1999 were obtained from the CDC [30-33] or the individual state Departments of Health [34-37]. When a discrepancy occurred, the data from the source with the higher number of cases reported for that year was used.

## Results

A total of 2,518 cases admitted to hospital for pertussis during the four-year period were identified (Table 1). Females accounted for 51% of the admissions. The mean age was 2.3 years (range: 0 – 83 years), but 90% of cases were younger than 1 year (median: <1 year) and most of the rest (8%) were for children. Admission via the emergency department was noted in 45% and 3% spent time in an observation unit prior to hospitalization.

**Table 1:** Summary of hospital stay results for pertussis by age group

	**Infants**	**Children**	**Adolescents & Adults**
Pertussis cases: n (%)	2,266 (90)	191 (8)	61 (2)
Age range (years)	<1	1 – 11	12 – 83
Mean age (years)	*	3	40
Males (%)	50	37	46
Emergency Department (%)	45	40	45
Observation Unit Stay (%)	0	0	3
Mean Total Length of Stay (days)	6	4	3
Mean Total Cost (per stay)	$9,580	$4,729	$5,310

* Age in months cannot be provided as databases used for this analysis do not provide those data.

**Figure 1:**
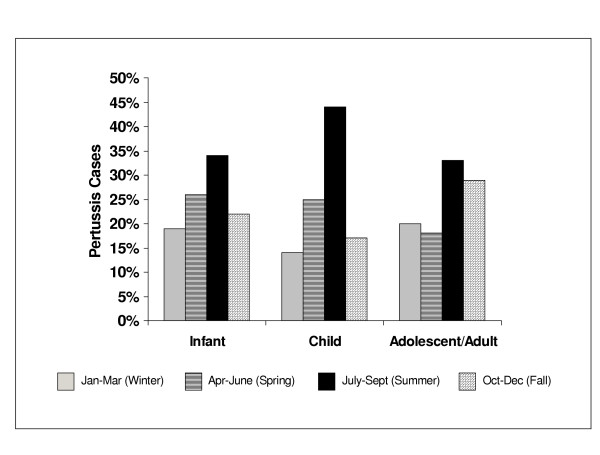
Admissions for pertussis by age group for years 1996 – 1999 by months of hospitalization.

The third quarter (July through September) accounted for more admissions (33%) than the other quarters. For infants and children, spring and summer months had the most admissions, while among older patients, summer and fall were more active (Figure 1).

The mean LOS was 6 days (median: 3.5, range: 1 – 110 days) and 13% of patients spent a portion of their stay in an intensive care unit. The mean cost of a hospital stay was estimated to be $9,130 per person (median: $4,600, range: $520 – $507,697). The cumulative cost over four years for all hospitalized pertussis cases examined in this study was estimated to be $29.4 million. Medicaid, a health insurance plan jointly funded by federal and state governments for those with low income who qualify, was the responsible payer for the majority (54%) of hospitalized cases. Managed care organizations were the next largest payer group, responsible for almost one third (31%) of the hospital stays.

Pneumonia was coded in 6% of cases and apnea or respiratory failure in 4%. Convulsions were recorded for 1%. Encephalopathy was recorded in less than 1% (0.3%) of all cases. The case fatality rate was less than 1% (n = 7). Six of the deaths occurred among infants, and the other was a patient aged 75 years. The vast majority (99%) went home (6% with other home health care services) and the rest (1%) returned to long-term or residential care facilities.

**Figure 2:**
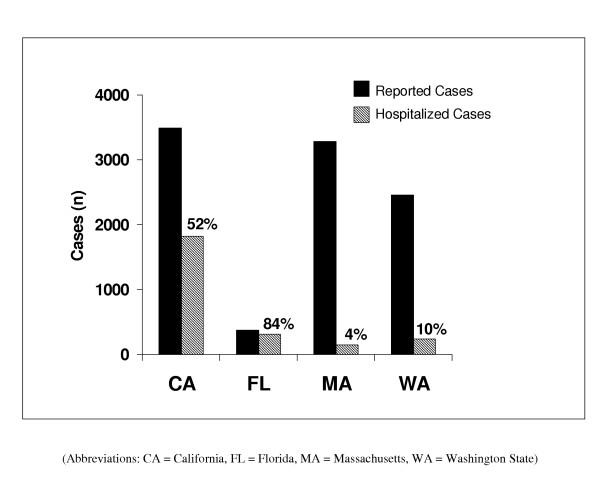
Number and proportion of hospitalized compared to all reported pertussis cases by state for 1996 – 1999.

During the years 1996 to 1999, the four states reported 9,605 pertussis cases, representing one third of all cases reported in the US over that period [30-37]. Hospitalized cases were 26% of those reported in these four states. This proportion varied substantially by state (Figure 2).

### Infants

Those less than one year of age accounted for 90% (n = 2,266) of all pertussis admissions. Males and females were represented equally. As precise age data are not available in these data sets, the distribution by month of age cannot be determined. A stay in an observation unit prior to admission was noted in 3% of cases and 45% were treated in the emergency department prior to admission. The majority (59%) of hospitalizations for infants occurred in the months of April through September. Pneumonia was coded in 12% of infant cases; apnea, respiratory distress or failure in 16%; convulsions in less than 1%, and encephalopathy in 0.2%. The mean LOS was 6 days (median: 4.5, range: 1 – 110 days) and 14% of all infants spent an average of 8 days in an intensive care nursery or pediatric intensive care unit. The cost per hospital stay averaged $9,580 (median: $4,670, range: $515 – $496,712). The inpatient case fatality rate was less than 1%. All survivors went home, of whom 6% were referred for home health care services, with less than 1% discharged on continuing intravenous drug therapy. Medicaid was the responsible payer for 57% of the hospital stays.

### Children

Children, aged 1 through 11 years, represented 8% (n = 191) of the admissions. The majority was female (63%) and 40% were seen in the emergency department prior to admission. The mean age for children was 3 years (median: 2). The average stay was 3.7 days (median 3, range 1 – 10 days); 15% had pneumonia coded; 5% convulsions and 8% apnea, respiratory distress or failure. Admissions for children were more in the summer months, (58%) in the months of June through September. The cost per hospital stay, estimated at $4,729 (median: $4,400, range: $537 – $49,945), was the lowest of the three age groups. This was not merely a reflection of the shorter stays but also a lower average per diem cost ($1,233) compared to infants ($1,347) and adolescents/adults ($1,612). No children died during hospitalization; all were discharged home (4% with home health care services). Medicaid (42%) and managed care organizations (39%) were responsible for most of the hospital stays.

### Adolescents and adults

**Figure 3:**
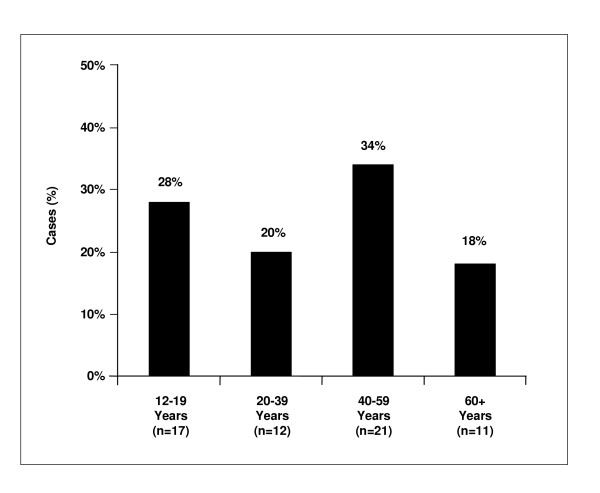
Age distribution of adolescent/adult hospitalized pertussis cases.

Those aged 12 and older represented only 2% (n = 61) of the hospitalized cases. Slightly more than half (54%) of them were female; mean age 40 years (median: 40, range: 12 – 83 years, Figure 3). Adults (mean age 50 years, median 49 years, range: 22 – 83) had the highest rate (48%) of admissions via the emergency department while the 17 adolescents (mean age: 13.5 years, range: 12–18 years) had the lowest rate (29%) of all groups, but 17% spent time in an observation unit prior to hospitalization.

One third (32%) of these admissions occurred between July and September; another 28% were admitted from October through December. The mean LOS was 3.4 days (median: 3, range: 1–11 days) and did not vary appreciably by age (adolescents, mean: 3, median: 3.2 days; adults, mean 3.4, median 3 days). In the small sample of adolescents, no one spent time in an intensive care unit, whereas 11% of the adults did, an average of one day. Pneumonia was noted in 11%; respiratory distress or failure in 8%; and convulsions in 2%. Among these patients, only one, aged 75 years died during hospitalization. The average cost per stay was $5,310 (median: $4,293, range: $685 – $27,625). All survivors went home, with 4% referred for home health care. Managed care organizations were the responsible payer for 43% of the patients in this group, the largest primary payer for both adolescents and adults. In the adult group, Medicare was the primary payer for 18% of the cases. Medicare is the federally funded health insurance program for those aged 65 years and older, as well as some younger patients who qualify because of disabilities.

## Discussion

This study shows that hospitalization is a common consequence of pertussis severe enough to be diagnosed and reported – one quarter of the cases of pertussis in these four states were admitted. Almost all of these patients admitted with pertussis survived and returned home, yet these results show that the economic consequences of pertussis at any age can be considerable. This has budgetary implications for both the public and private sector, as the responsible payers for the majority of cases were Medicaid or managed care organizations. Caring for patients with pertussis who require hospitalization generates millions of dollars in health care expenses and yet this substantial amount is a conservative estimate as it reflects but one aspect of care. As the focus of this analysis was limited to examining direct medical care costs incurred during hospitalization, post-acute care and outpatient care costs were not included in the estimates. Most patients will be treated as outpatients and will use other health care services, such as office and emergency department visits, and antibiotics. Neither those costs nor indirect costs have been considered in this analysis; however, a study examining pertussis cases in one New York county over a six year period from 1989–1994 [38] reporting inpatient and outpatient care costs noted that the cost of hospitalization represented one of the two largest contributing costs associated with pertussis. Furthermore, this study and another [39] by the same author, also examined indirect costs related to pertussis and found that work-related (e.g., lost work days, decrease in productivity) costs were responsible for more than 60% of the overall cost of pertussis when both direct and indirect costs were considered. Thus, while the cost of hospital care provides a key component of pertussis-related care costs and must be considered in any economic analysis of the disease, it does not convey fully the economic impact of the disease.

The sample size for adolescents and adults in this analysis was small. There are several possible reasons for this. Pertussis in older patients often presents in an atypical manner without the characteristic whoop [2,6,7,13], which may contribute to misdiagnosis. In such circumstances, those cases would not be identified by the pertussis-related ICD-9 codes and therefore not captured, or counted, for this analysis. It is more likely, however, that the severity of pertussis in older age groups does not warrant inpatient management as often as required for infants and young children. In the 1993 pertussis outbreak in Cincinnati [5], 84% of the hospitalizations were among those less than 12 months old.

Pneumonia was noted as a complication in 6% for all cases and in 12% of infants in our analysis. For seizures, we report a 1% rate for all cases and <1% for infants; 0.3% and 0.2% rates of encephalopathy in all cases and among infants, respectively. These rates appear comparable to those reported by the CDC based on data from 1997–2000 [40]. Among all reported cases, pneumonia occurred in 5.2% of pertussis cases and in 11.8% of infants < 6 months. Seizures were reported by the CDC in 0.8% for all reported pertussis cases and 1.4% for infants; encephalopathy among 0.1% for all cases and 0.2% among infants. Rate reporting for other age groups is not done in a comparable manner as the range of ages within the child and adolescent/adult group differ; however, it does appear that the rates of 15% among children and 11% among adolescents/adults for pneumonia coded in our analysis are higher than rates reported for all ages over age one year by the CDC. The reason for this discrepancy is unknown. It may reflect an error in coding or that this analysis is based only on more severe hospitalized cases; whereas, the CDC is reporting complications rates for all reported cases of pertussis.

Using data from any large administrative database raises concern about potential coding errors. The coding error rate for pertussis in the state databases is unknown, but it is not uncommon for an infectious disease to be identified with less specific codes. This has been reported for other conditions, such as pneumonia [41]. Therefore, it is possible that pertussis cases could have been coded to more generic respiratory conditions, such as unspecified upper respiratory infection (ICD-9 code 465.9). In 1999, there were 5,449 hospitalizations in these databases coded with this non-specific principal diagnosis. It has been reported that 39% of university students [6] who were ultimately determined to have pertussis were first diagnosed by their primary care provider as having an upper respiratory tract infection. Another 48% in that study were misdiagnosed with bronchitis.

A large proportion of the reported cases in some of the states were hospitalized, possibly suggesting that only the most severe cases are being reported; however, this analysis showed a large disparity in these proportions among the four states. The reason for the disparity is not known and can not be determined based on the data available. It is more likely, however, that there are multiple factors contributing the variability seen in the rates of hospitalization among states rather than a differing clinical profile or admitting threshold. Among these factors could be how pertussis cases are reported in each state, the number of cases of infant versus adolescent/adult disease for a state, or that many are not being diagnosed correctly and therefore go unreported.

Based on the results of our analysis and those of others [2,8,11,12,14,15,21], it is evident that pertussis is neither a disease limited to childhood nor one that has been conquered through current immunization programs. The rise in pertussis incidence in the past several years highlights the growing vulnerability of those beyond the protection afforded by childhood immunization. While newer acellular pertussis vaccines may permit extension of immunity to an older population, until very recently booster doses of pertussis-containing vaccines were not available for those over the age of seven years [1].

While efforts have been made to make the clinical community more aware of the increasing occurrence of this disease in older patients, it has been shown that pertussis often goes unrecognized, undiagnosed and underreported in adolescents and adults [7,8,11,19]. Thus, the burden of illness reflected in the results from this study, particularly with regard to older patients may be much lower than in reality.

Clearly, the impact of immunization on the disease burden and costs will depend largely on the extent of herd immunity attained and this is not yet securely known for any particular level of coverage.

## Conclusion

The results reported in this analysis, albeit limited to one level of management, illustrate how costly it can be to manage pertussis when hospitalization is required. These results provide decision makers concerned with the economic aspects of preventing and treating pertussis with recent and relevant cost estimates for a substantial cost component of managing pertussis; however, to assess the cost of an expanded vaccination program, all aspects of the economic burden of caring for patients with pertussis, including indirect costs, will need to be considered.

## Competing interests

Caro Research, of which Judith A. O'Brien and J. Jaime Caro are shareholders, received an unrestricted grant from Aventis Pasteur SA for this work. Relevant staff from Aventis Pasteur SA were allowed to review and comment on this manuscript but were explicitly forbidden from exerting any editorial control. The authors are employees of Caro Research. Expenses for travel to present the findings at the 2004 International Congress of Infectious Disease were reimbursed.

## Authors' contributions

JAO conceived of and designed the study, performed the analysis, and participated in drafting the manuscript. JC participated in the design of the study and participated in drafting the manuscript. All authors read and approved the final manuscript.

## Pre-publication history

The pre-publication history for this paper can be accessed here:


